# Prognostic value of red blood cell distribution width and D‐Dimer in diffuse large B‐cell lymphoma: Systematic review and meta‐analysis

**DOI:** 10.1002/cnr2.1936

**Published:** 2023-11-24

**Authors:** Maryam Rahchamani, Mohammad Sedghi, Ali Hakimi, Mahdi Nejatianfar, Tohid Javaheri, Reza Tavakoli, Ramtin Ahmadi, Mansoureh Makarem, Nazanin Azmi‐Naei, Saeid Zahmatkesh Sangani, Neda Kamandi, AmirMohammad Soleimanian, Rasoul Shavaleh, Molood Foogerdi, Kazem Rahmani

**Affiliations:** ^1^ Department of Internal Medicine, Faculty of Medicine Tehran University of Medical Sciences Tehran Iran; ^2^ Molecular and Cellular Biology, Department of Biology, Yadegar‐e‐Imam Khomeini (RAH) Shahr‐e‐Ray Branch Islamic Azad University Tehran Iran; ^3^ Department of Clinical Biochemistry Mashhad University of Medical Sciences Mashhad Iran; ^4^ Department of Research and Development Organic Chemistry Hila Pharmaceutical Co Mashhad Iran; ^5^ Department of Genetics, Young Research and Elites Club Islamic Azad University, Mashhad Branch Mashhad Iran; ^6^ Department of Radiology Arak University of Medical Sciences Arak Iran; ^7^ Cellular and Molecular Islamic Azad University of Mashhad Mashhad Iran; ^8^ Health Vice‐Chancellor Torbat‐e Jam Faculty of Medical Sciences Torbat‐e Jam Iran; ^9^ Department of Epidemiology, School of Public Health Shahroud University of Medical Sciences Shahroud Iran; ^10^ Faculty of Medicine Mashhad University of Medical Sciences Mashhad Iran; ^11^ Department of Epidemiology, School of Public Health Iran University of Medical Sciences Tehran Iran; ^12^ Department of Emergency Medicine, Faculty of Medicine Birgand University of Medical Sciences Birjand Iran

**Keywords:** D‐Dimer, DLBCL, non‐Hodgkin's lymphoma, prognosis, RDW

## Abstract

**Background:**

The significant role of red blood cell distribution width (RDW) and D‐Dimer as prognostic factors in patients with some blood malignancies has been reported recently.

**Aim:**

We designed and performed a meta‐analysis to investigate the prognostic roles of RDW and D‐Dimer in subjects with diffuse large B‐cell lymphoma (DLBCL).

**Materials and Methods:**

We systematically reviewed PubMed‐Medline, SCOPUS, EMBASE, Web of Science Core Collection, and Google Scholar up to the present to look for publications on prognostic effects of RDW and D‐Dimer in DLBCL patients. For investigation of the associations between RDW and D‐Dimer with the overall survival (OS) and progression‐free survival (PFS) of the DLBCL cases, hazard ratio (HR) with 95% confidence intervals (CIs) was used.

**Results:**

We included 13 eligible studies in the present meta‐analysis. The results of pooled analysis showed that increased levels of RDW was related to poor OS (HR = 2.01, 95% CI: 1.62–2.48, *p* value <.01, *I* ^2^ = 0%) and poor PFS (HR = 1.52, 95% CI: 1.24–1.85, *p* value <.01, *I* ^2^ = 16%) among the DLBCL patients. Similarly, a significant relationship was found between increased D‐Dimer and poor OS (HR = 2.30, 95% CI: 1.03–5.14, *p* value <.05, *I* ^2^ = 95%) of the DLBCL patients as well. In addition, there was no significant heterogeneity in OS (*p* value *H* = 0.65) and PFS (*p* value *H* = 0.31) related to RDW among studies included in the meta‐analysis.

**Conclusion:**

Our finding clearly confirmed that elevated RDW levels and D‐Dimer were associated with adverse OS and PFS in DLBCL.

## 1 INTRODUCTION

Among common types of non‐Hodgkin's lymphoma, diffuse large B‐cell lymphoma (DLBCL) accounts for approximately 35% of lymphoma cases in western countries.^1^, ^2^, ^3^ Most often, chemoimmunotherapy with standard regimens such as R‐CHOP (rituximab, cyclophosphamide, doxorubicin, and vincristine prednisone) is the treatment for DLBCL patients. However, prognosis of these patients is highly heterogeneous and it is very difficult to predict the final outcome of the disease in the patients. In addition, about 40% of DLBCL patients experience relapse or are resistant to treatment.^4^, ^5^

In the recent years, several prognostic factors such as Hemoglobin, Lymphocyte/Monocyte Ratio, Beta‐2 microglobulin, and Neutrophil/Lymphocyte Ratio have been proposed to project the survival rate of DLBCL patients which can be easily performed in a common laboratory.^6^, ^7^, ^8^, ^9^ Nevertheless, easier and more available factors with high sensitivity are required for prediction of prognosis of DLBCL patients. Red blood cell distribution width (RDW) is considered a systemic inflammatory response marker that can be easily evaluated through the Complete Blood Count (CBC) test and has acceptable sensitivity in many diseases such as cardiovascular and autoimmune diseases and sepsis.^10^, ^11^, ^12^, ^13^ D‐Dimer as a sensitive index of the process of fibrin formation and destruction is widely used in the deep venous thrombosis detection, intravascular coagulation, sickle cell anemia, and myocardial infarction.^14^, ^15^, ^16^, ^17^, ^18^ Recent studies introduced that tumor‐related degradation products for the coagulation and fibrinolytic system, such as D‐Dimer, can be used as outcomes prediction for tumor.^19^ Furthermore, scientific evidence recently revealed the association of RDW and D‐Dimer with many cancers such as breast cancer,^20^, ^21^, ^22^ lung cancer,^23^, ^24^ blood cancers,^25^, ^26^, ^27^ prostate cancer,^28^ and other malignancies.^29^, ^30^, ^31^, ^32^ However, the prognostic roles of RDW and D‐Dimer in cancer and its possible mechanism in tumor progression are being discussed. Besides, several studies indicated that D‐Dimer had a possible role in proliferation of cancer cells, adhesion, and angiogenesis, which may bring about malignant tumors growth. Cancer patients usually face hypercoagulable states that increase the risk of embolism. Hence, D‐Dimer levels have prognostic role and are associated with survival of patients with cancer.

Considering the differences between the studies regarding the sample size, study design, and also the existence of controversy among their results, a comprehensive and complete investigation of the prognostic role of RDW and D‐Dimer in DLBCL patients seems necessary. This study is the first systematic review and meta‐analysis designed to investigate the prognostic role of RDW and D‐Dimer in DLBCL patients.

## 2 MATERIALS AND METHODS

We utilized the checklist of meta‐analysis of observational studies^33^ and preferred reporting items for systematic reviews and meta‐analyses (PRISMA) standard^34^ to perform this study. The protocol was registered with the number of CRD42023417907 at the international prospective register of systematic reviews database (PROSPERO).

### 2.1 Search strategy

A systematic search in PubMed, Medline, SCOPUS, Web of Science Core Collection, EMBASE, and Google Scholar databases was carried out to find the records from inception to present. Using the keywords including RDW, red blood cell distribution width, DLBCL, diffuse large B‐cell lymphoma, lymphoma diffuse, large B‐cell, fibrin fragment D, D‐Dimer, D‐Dimer fibrin, and D‐Dimer fragments, two researchers (KR and RS) searched in the mentioned databases separately and blindly. Inconsistencies in some articles were also solved by other researchers (NAN, MR, MF, and NK). Finally, duplicates were identified by title of the papers, authors' names, and journals' names.

### 2.2 Eligibility criteria

**FIGURE 1:**
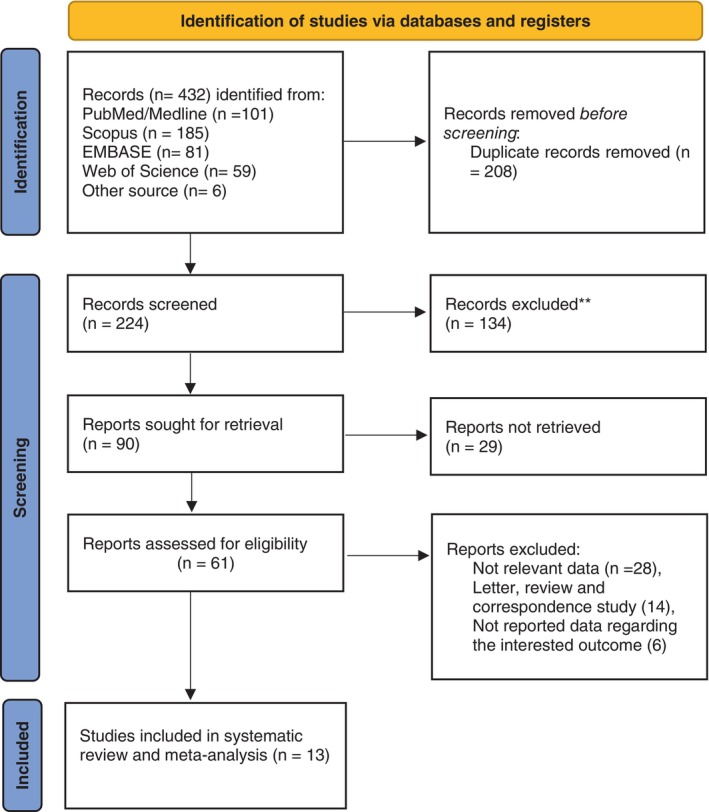
PRISMA flow diagram illustrating the process of selection studies in the meta‐analysis.

We included all the observational studies which examined the role of RDW and D‐Dimer as prognostic factors in DLBCL cases and also published in English without time and place restriction. On the other hand, studies such as case reports, case series, letters, and correspondence studies were among our exclusion criteria (Figure 1). The articles conducted regarding autoimmune diseases, Immunosuppression, patients with mental disorders, and those undergoing dialysis were excluded as well. In addition, the studies with lack of sufficient information on overall survival (OS) as well as progression‐free‐survival (PFS) of DLBCL subjects were excluded from the review process.

### 2.3 Data extraction and quality assessment

Articles were screened independently by two researchers (RT and RM) (according to Figure 1) and disagreements resolved through discussions with other researchers. The extracted information included author's first name, study location, study type, type of marker investigated, year of publication, type of survival analysis performed, mean follow‐up period, sample size, and a summary of the cut‐off points for the markers investigated. OS is usually the time length from the beginning of medical treatment until outcome occurrence (death whether or end of the follow‐up period of the patient). PFS is the duration when the treatment is started until tumor progresses or death happens for any reason. In order to evaluate quality of the articles, two researchers (MR and AH) independently used the checklist of Newcastle Ottawa Scale (NOS).^35^ The articles with a total score of ≥7 were considered as high‐quality.

### 2.4 Statistical analysis

We used R version 4.3.0 for performing the statistical analysis. *I* ^2^ and *χ* ^2^ statistics were applied for assessing the heterogeneity of the studies and the significance level for heterogeneity between the studies was considered *I* ^2^ > 50% and *p* value <.1. We used a random effect model so as to compute pooled hazard ratio with 95% confidence interval and the Inverse Variance method was applied to weight the studies. The researchers also used funnel plot and Eggers' test to check the publication bias in the studies and the asymmetric funnel plot was considered as a possible publication bias. Sensitivity analysis was utilized to examine the heterogeneity source between the records. The significance level in this study was considered <.05.

## 3 RESULTS

A total of 13 studies^1^, ^6^, ^7^, ^16^, ^19^, ^36^, ^37^, ^38^, ^39^, ^40^, ^41^, ^42^, ^43^ were included in our study (PRISMA flow diagram) based on the searched databases. Overall, eight studies^1^, ^6^, ^7^, ^37^, ^39^, ^40^, ^41^, ^43^ focused on the role of RDW and the remaining five studies^16^, ^19^, ^36^, ^38^, ^42^ focused on D‐Dimer levels in DLBCL patients.

There were some studies that met the inclusion criteria but were not included in the meta‐analysis due to the lack of relevant information or the incompleteness of reporting of the studied indicators.^44^, ^45^, ^46^, ^47^, ^48^ The included studies were published from 2015 to present. All included articles were retrospective cohorts. Eight studies were conducted in China^7^, ^16^, ^19^, ^36^, ^38^, ^39^, ^40^, ^43^ and others were conducted in Croatia,^41^ Iraq,^37^ Japan,^42^ Peru,^1^ and Spain.^6^ The total sample size of the meta‐analysis was 3972 people (1252 and 2720 patients in the studies were related to prognostic roles of D‐Dimer and RDW in DLBCL patients, respectively) varying from 81 to 992 people in the included studies. Other details of the included studies are shown briefly in Table 1.

**TABLE 1:** Summary of studies on the prognostic factors such as RDW and D‐Dimer among patients with DLBCL.

First author	Type of study	Country	Year	Type of marker	Description	Type of analysis	Follow up time (month)	Sample size	Age	Gender ratio (male:female)	Treatments received	IPI score
Tanaka et al.^42^	Retrospective	Japan	2018	D‐Dimer	Cut‐off value of D‐Dimer <1.0 μg/mL for LD and ≥1 μg/mL for HD	OS	30.2 (0.1–162)	391	Median: 70 (30–92)	1.184	Pretreatment	Low risk: 192 (49.1%) High risk: 199 (50.9%)
Geng et al.^19^	Retrospective	China	2019	D‐Dimer	Cut‐off value of D‐Dimer 0.92 μg/mL	OS	14 (1–82)	113	Mean: 52 (17–82)	1.132	‐	‐
Duan et al.^36^	Retrospective	China	2022	D‐Dimer	‐	PFS	‐	186	Age <60: 96 (51.62%) Age ≥60: 90 (48.38%)	1.22	R‐CHOP/CHOP	Low risk: 103 (55.38%) High risk: 83 (44.62%)
Liu et al.^16^	Retrospective	China	2018	D‐Dimer	Cut‐off value of D‐Dimer ≥1.6 μg/mL	OS	33 (1–86)	254	Median: 55 (15–82)	1.29	Pretreatment	Low risk: 91 (35.8%) Intermediate risk: 100 (39.4%) High risk: 36 (24.8%)
Huang et al.^38^	Retrospective	China	2021	D‐Dimer	Cut‐off value of D‐Dimer ≥1.4 μg/mL For HD and D‐Dimer <1.4 for LD	OS, PFS	22.13 (2.73–89.07)	308	Median: 56 (14–86)	1.61	Pretreatment	Low risk: 124 (40.25%) Low‐intermediate risk: 67 (21.75%) High‐intermediate risk: 65 (21.11%) High risk: 52 (16.89%)
Beltran et al.^1^	Retrospective	Peru	2019	RDW	High RDW (>14.6%) and normal RDW (11.6%–14.6%)	OS	67.2	121	Age ≥60: 78 (67%)	0.76	R‐CHOP	Low risk: 68 (58%) High risk: 49 (42%)
Bento et al.^6^	Retrospective	Spain	2019	RDW	RDW ratio >0.96 according to the new prognosis score	OS, PFS	55 (12–185)	992	Median: 64 (18–91)	1.018	R‐CHOP	‐
Hasan and Elmeshhedany^37^	Retrospective	Iraq	2021	RDW	High RDW (≥14.85%) and Low RDW (<14.85%)	OS, PFS	35 (3–133)	136	Median: 51 (15–81)	2.09	R‐CHOP	Poor: 28 (20.59%) Good: 73 (53.68%) Very good: 35 (25.73%)
Periša et al.^41^	Retrospective	Croatia	2015	RDW	Elevated RDW > 15%, Normal RDW ≤ 15%	OS, ESF	22 (8.5–37.5)	81	Median: 64 (52.5–72.5)	0.557	R‐CHOP	Low risk: 46 (56.8%) High risk: 35 (43.2%)
Li et al.^39^	Retrospective	China	2022	RDW	High RDW (≥13.40%), Low RDW (≤12.60%) and Medium RDW (13.4% > RDW > 12.6%)	OS, PFS	35 (1–82)	179	Median: 58 (24–88)	1.46	Methotrexate‐based combination immunochemotherapy	Low risk: 115 (77.71%) High risk: 33 (22.29%)
Zhou et al.^43^	Retrospective	China	2017	RDW	High RDW (≥14.1%) and Low RDW (<14.1%)	OS, PFS	42 (6–120)	161	Median: 59 (18–80)	1.30	R‐CHOP and R‐CHOP‐like	Low risk: 93 (57.77%) Intermediate risk: 55 (34.16%) High risk: 13 (8.07%)
Li et al.^40^	Retrospective	China	2019	RDW	High RDW (>14.35%) and Low RDW (≤14.35%)	OS, PFS	21.3 (0.80–126.93)	349	Age >60: 146 (41.84%)	1.21	R‐CHOP and CHOP	Low risk: 248 (71.07%) High risk: 101 (28.93%)
Chen et al.^7^	Retrospective	China	2022	RDW	High RDW (≥14.50%) and Low RDW (<14.50%)	OS, PFS	85.2 (0.5–179.6)	998	Median: 53 (7–83)	1.23	R‐CHOP and R‐CHOP‐like	Low risk: 552 (55.30%) Low‐intermediate risk: 203 (20.3%) High‐intermediate risk: 156 (15.6%) High risk: 87 (8.7%)

Abbreviations: EFS, event free survival; HD, high D‐Dimer; LD, low D‐Dimer; NOS, Newcastle–Ottawa Quality Assessment Scale; OS, overall survival; PFS, progression free survival.

### 3.1 Overall survival in RDW and D‐Dimer

**FIGURE 2:**
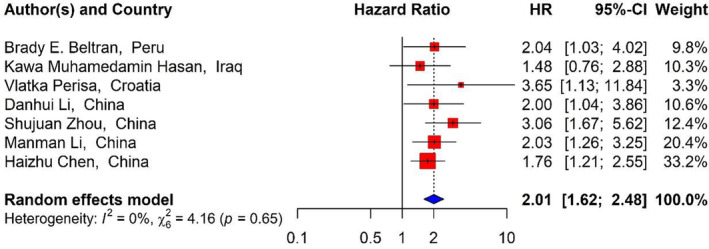
Forest plots of studies evaluating the relationship between RDW and OS.

**FIGURE 3:**
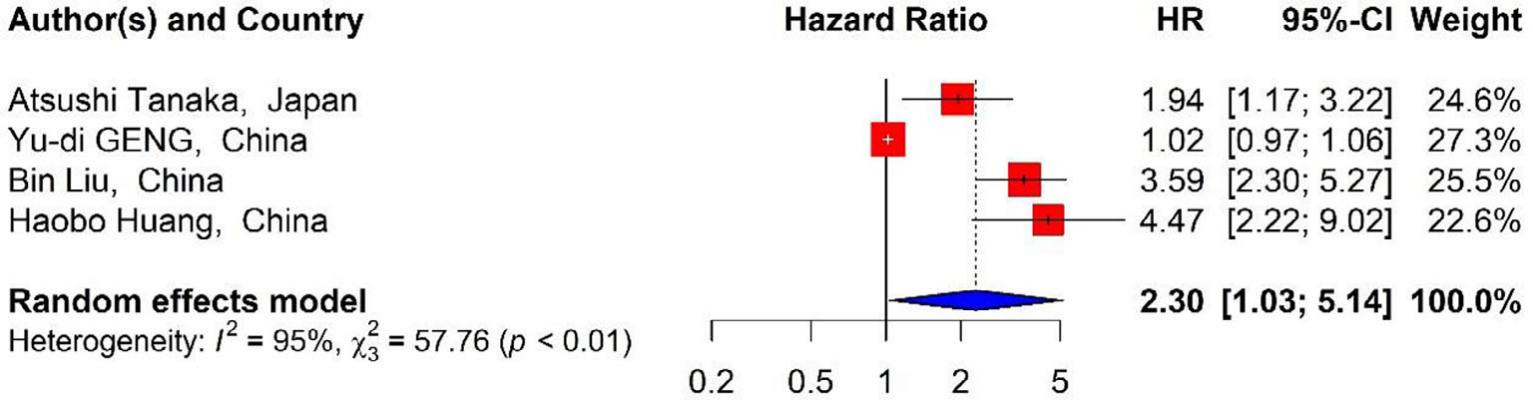
Forest plots of studies evaluating the relationship between D‐Dimer and OS.

To examine the relationship between the RDW in DLBCL patients and OS, seven studies^1^, ^7^, ^37^, ^39^, ^40^, ^41^, ^43^ with 1728 patients were included. Our findings showed that RDW levels had relationship with OS. The pooled analysis presented a significant association between increased RDW and adverse OS of the patients (HR_Pooled_ = 2.01, 95% CI: 1.62–2.48, *p* value <.01) (Table 1) (Figure 2). Examining the correlation between the D‐Dimer and OS among the patients with DLBCL was performed by four studies^16^, ^19^, ^38^, ^42^ with 1066 patients and D‐Dimer levels correlated with poorer OS. The combined results also demonstrated that increased D‐Dimer was associated with worse OS of the patients (HR_Pooled_ = 2.30, 95% CI: 1.03–5.14, *p* value <.05) (Figure 3).

### 3.2 Progression‐free‐survival and RDW

**FIGURE 4:**
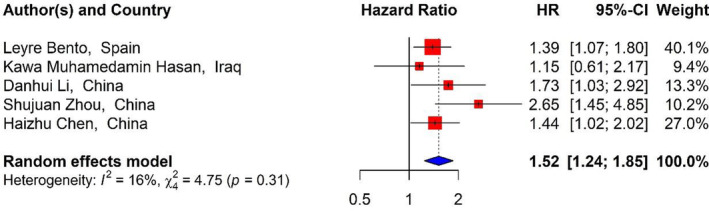
Forest plots of studies evaluating the relationship between RDW and PFS.

Five studies^6^, ^7^, ^37^, ^39^, ^43^ with 2169 patients reported the relationship between the RDW levels and PFS. In this study, the pooled HRs showed that increased RDW correlated with worse PFS in the DLBCL patients (HR_Pooled_ = 1.52, 95% CI: 1.24–1.85, *p* value <.01) (Figure 4).

### 3.3 Sensitivity analysis

With respect to the sensitivity analysis, the results did not show significant difference between the included articles regarding the relationship between RDW and D‐Dimer and OS, and the relationship between RDW and PFS among the DLBCL patients (supplementary file Figures 1–3).

### 3.4 Publication bias

**FIGURE 5:**
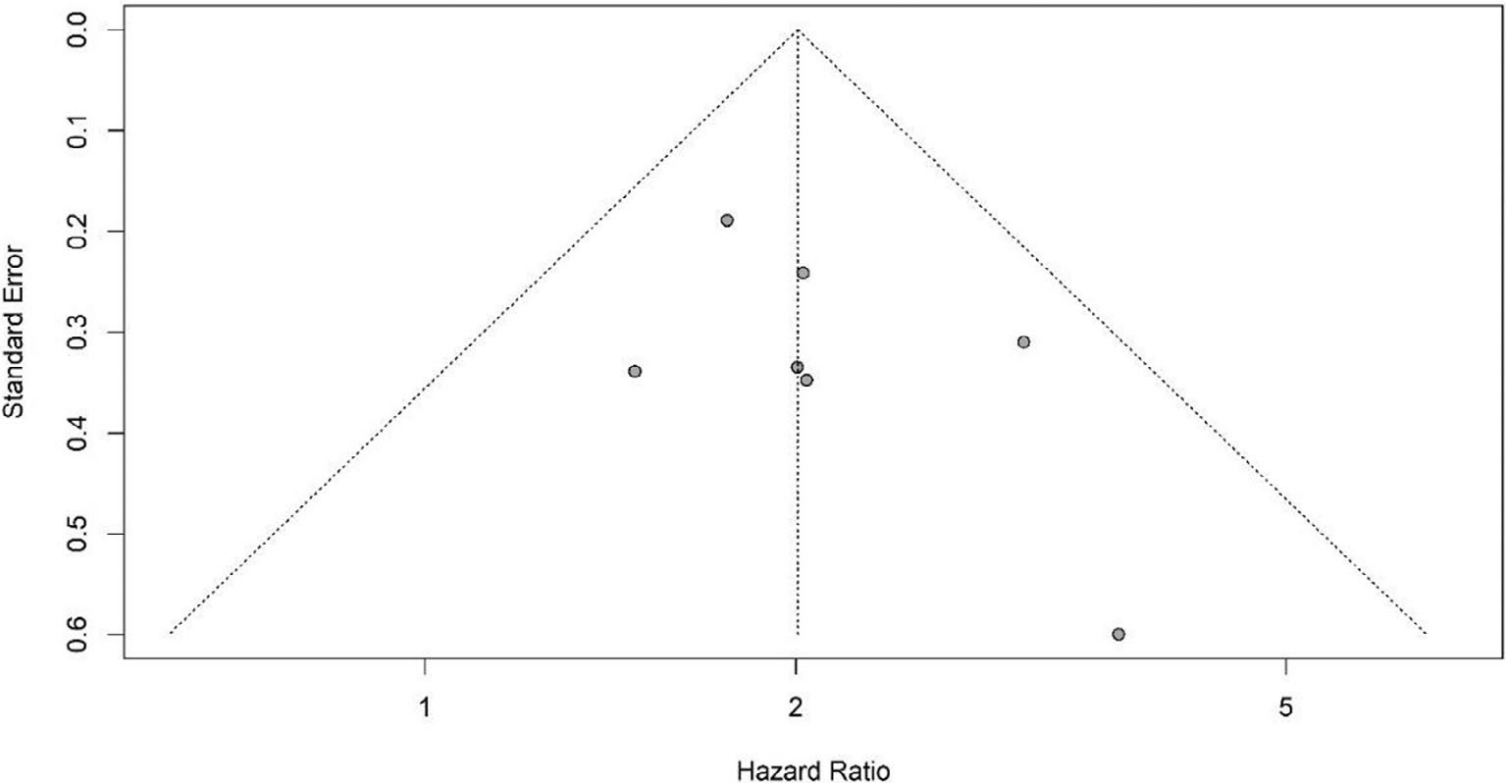
Funnel plot with pseudo 95% confidence limits for RDW and OS.

**FIGURE 6:**
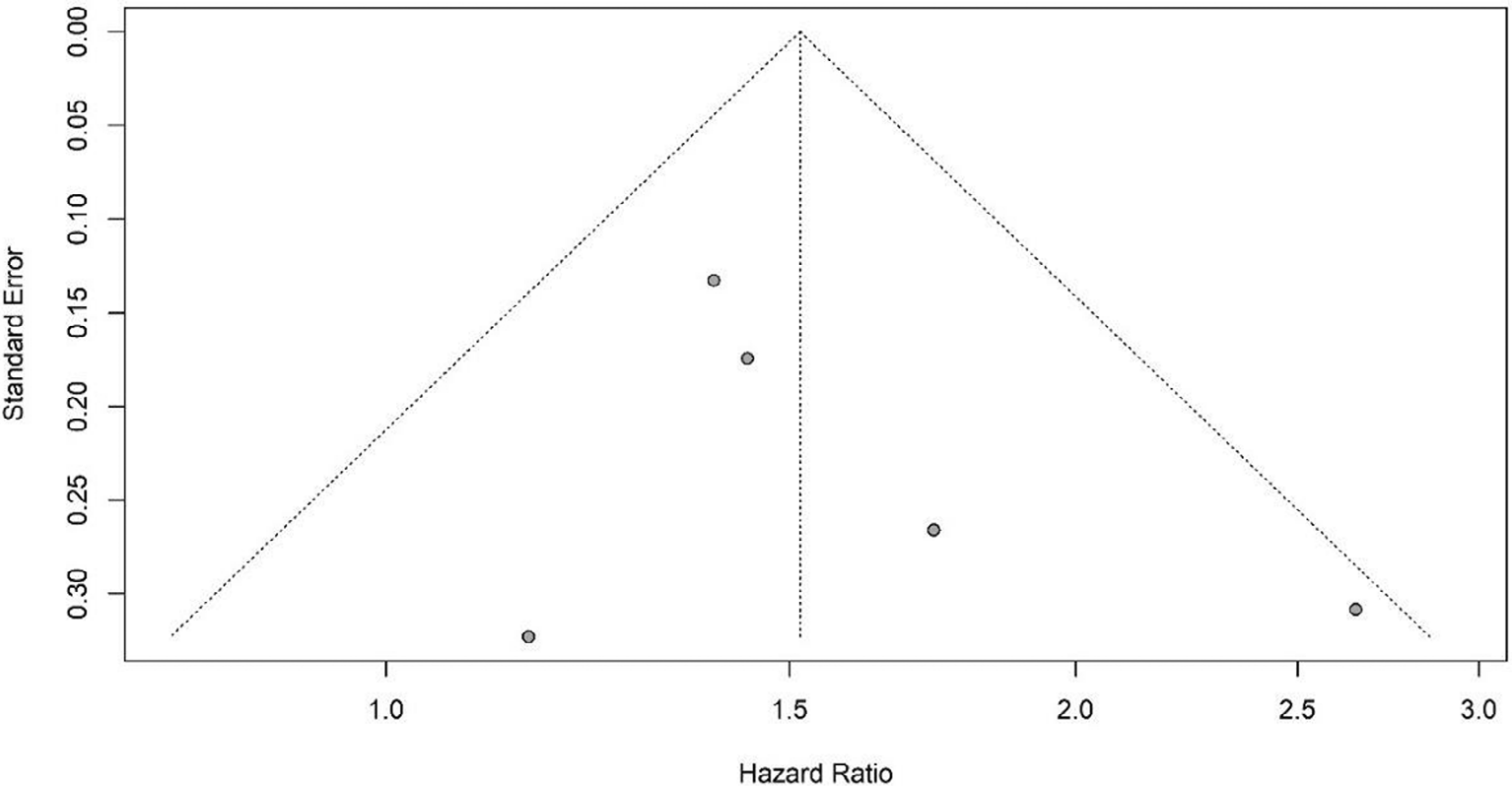
Funnel plot with pseudo 95% confidence limits for RDW and PFS.

**FIGURE 7:**
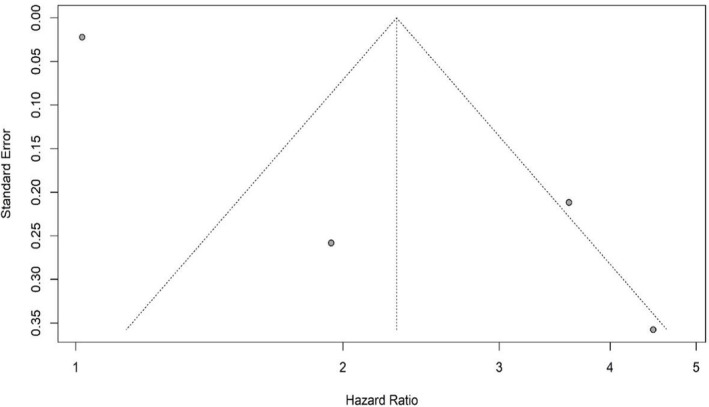
Funnel plot with pseudo 95% confidence limits for D‐Dimer and OS.

By checking the symmetry of the Funnel Plot, the results showed that the Funnel Plot of the studies included in the meta‐analysis that investigated the prognostic role of RDW in the OS and PFS of DLBCL patients had relatively symmetry (Figures 5 and 6). The results of Eggers' test also showed that there was no significant publication bias in the studies included in the RDW meta‐analysis in patients' OS (*t* = 1.353, *p* value = .234) and PFS (*t* = 0.939, *p* value = .418). Moreover, the Funnel Plot of the studies that investigated the prognostic role of RDW in the OS of DLBCL patients had relatively symmetry (Figure 7) and Eggers' test results also indicated the absence of significant publication bias between the included studies (*t* = 4.034, *p* value = .06).

## 4 DISCUSSION

The prognostic roles of RDW and D‐Dimer were explored in subjects with DLBCL. We observed that high RDW and D‐Dimer were two prognostic factors related to OS and PFS among DLBCL cases. Recently, evidence has been confirmed the relationship between RDW and poor prognosis in various cancers.^49^, ^50^ Furthermore, increased RDW was associated with worse prognosis in blood malignancies such as chronic myeloid leukemia,^51^ chronic lymphocytic leukemia,^52^ NK/T lymphoma,^53^ multiple myeloma,^54^ and DLBCL.^40^

Inflammation is a pivotal factor which plays a role in progression of tumor and is known as one of its prominent features.^25^, ^55^ In this study, the pooled analysis results revealed that high values of RDW were associated with poor PFS and OS. Although the main mechanism of the relationship between RDW and the prognosis of DLBCL patients has not been clearly and fully investigated, high RDW can be attributed to the disturbance in erythropoiesis and the changes in red blood cell maturation.^56^

Some findings have provided evidence that there is an association between RDW and some markers such as IL‐6, CRP (C‐reactive protein), TNF‐I and II (Tumor Necrosis Factor), TK (Thymidine Kinase), ESR (Erythrocyte Sedimentation Rate), and Ferritin which the accuracy and sensitivity in DLBCL patients are in a state of ambiguity.^13^ The relationship between RDW and malnutrition conditions might be explained by treatment with poor response and poor prognosis of patients who suffer from cancer.^41^ In addition, the disruption in iron absorption and metabolism mechanism observed in most cancers also contributes to increase the level of RDW,^57^ and increased RDW is considered as a turning point in the relationship between inflammation and worse prognosis of subjects who suffer from DLBCL. On the other hand, poor coagulation conditions are associated with poor prognosis and the outcomes such as venous thrombosis embolism (VTE) and disseminated intravascular coagulation (DIC).

Treatment of various cancers is associated with hypercoagulable states and according to the evidence, the changes in coagulation and fibrinolysis pathways have a great impact on cancer prognosis.^19^ Moreover, tumor‐related degradation products such as D‐Dimer as a prognostic factor of the final outcome should be used in all types of cancer.^20^, ^26^, ^29^ Some studies have indicated that elevated D‐Dimer values as a suitable factor in solid tumor patients is associated with poor prognosis^58^ and D‐Dimer decreases significantly after the first chemotherapy.^19^ In addition, it has been proved that there is an association between values of D‐Dimer and tumor progression, distant metastasis, and tumor volume.^59^, ^60^ However, our pooled analysis results showed that by increasing in the D‐Dimer values as a prognostic factor, we witnessed worsening OS in the DLBCL patients.

Since RDW and D‐Dimer usually reflect the presence of B symptoms, disease stage, and patient's performance status^43^ and considering the prognostic role of RDW and D‐Dimer in overall survival rate and disease‐free survival of DLBCL patients in this meta‐analysis, RDW and D‐Dimer can play a role in advanced‐stage tumors.

In terms of considerable heterogeneity between the studies included in the meta‐analysis, we realized that the study conducted by Geng et al.^19^ was the cause of heterogeneity between the studies. Although there was no difference between the methodology and the type of population studied by Geng with other studies, this could be due to the unknown type of treatment, the small sample size compared to other studies, and unbalanced grouping due to the type of study and heterogeneity between patients.

In this study, our findings determined the prognostic role of RDW and D‐Dimer in DLBCL patients. Since the values of RDW and D‐Dimer can be easily accessible in the patient tests at low cost, considering to these prognostic indicators would be helpful in the prognosis of DLBCL patients.

This meta‐analysis had some limitations. First of all, it can be pointed out that significant heterogeneity was observed in the analysis of D‐Dimer role in DLBCL patients, so the random‐effect approach was used in the analysis. Moreover, due to the limited number of studies on the role of D‐Dimer in DLBCL patients, it was not possible to analyze PFS in this meta‐analysis. Due to the difference between RDW cut‐off points in different studies, it was not possible to perform subgroup analysis based on RDW values.

## 5 CONCLUSION

This meta‐analysis indicated that high values of RDW and D‐Dimer were significantly associated with low prognosis and poor OS and PFS in DLBCL patients. Due to availability in routine laboratory tests and affordability, they could be helpful in disease progression and prognosis of DLBCL patients and also in clinical decisions. Nevertheless, doing further studies in this field seems necessary.

## AUTHOR CONTRIBUTIONS

**Maryam Rahchamani:** Data curation (equal); writing – review and editing (equal). **Mohammad Sedghi:** Funding acquisition (equal); investigation (equal). **Ali Hakimi:** Funding acquisition (equal); validation (equal); writing – review and editing (equal). **Mahdi Nejatianfar:** Funding acquisition (equal); validation (equal); visualization (equal). **Tohid Javaheri:** Resources (equal); software (equal). **Reza Tavakoli:** Resources (equal); software (equal). **Ramtin Ahmadi:** Visualization (equal); writing – review and editing (equal). **Mansoureh Makarem:** Funding acquisition (equal); investigation (equal); writing – review and editing (equal). **Nazanin Azmi‐Naei:** Software (equal); validation (equal); writing – review and editing (equal). **Saeid Zahmatkesh Sangani:** Data curation (equal); resources (equal); writing – review and editing (equal). **Neda Kamandi:** Data curation (equal); funding acquisition (equal); writing – review and editing (equal). **AmirMohammad Soleimanian:** Investigation (equal); resources (equal); writing – review and editing (equal). **Rasoul Shavaleh:** Conceptualization (equal); formal analysis (equal); software (equal); validation (equal); writing – original draft (equal). **Molood Foogerdi:** Project administration (equal); supervision (equal). **Kazem Rahmani:** Conceptualization (equal); formal analysis (equal); investigation (equal); methodology (equal); project administration (equal); validation (equal); writing – original draft (equal); writing – review and editing (equal).

## CONFLICT OF INTEREST STATEMENT

The authors have stated explicitly that there are no conflicts of interest in connection with this article.

## ETHICS STATEMENT

Not applicable.

## Supporting information


**Figure 1** Sensitivity analysis in association RDW and OS.
**Figure 2** Sensitivity analysis in association of D‐Dimer and OS.
**Figure 3** Sensitivity analysis in association of RDW and PFS.

**Table 1** Quality assessments of studies include in meta‐analysis.

## Data Availability

The original contributions presented in the study are included in the article/supplementary material, further inquiries can be directed to the corresponding author.
