# Supplementary material for: Prognostic value of red blood cell distribution width and D‐Dimer in diffuse large B‐cell lymphoma: Systematic review and meta‐analysis
Source: Cancer Rep (Hoboken). 2023 Nov 24;7(1):e1936. doi: 10.1002/cnr2.1936 (PMC10809198; doi:10.1002/cnr2.1936)
Supplement: Supplementary file 1 — Figure 1 Sensitivity analysis in association RDW and OS. Figure 2 Sensitivity analysis in association of D‐Dimer and OS. Figure 3 Sensitivity analysis in association of RDW and PFS. [file CNR2-7-e1936-s002.docx]

**Supplementary**


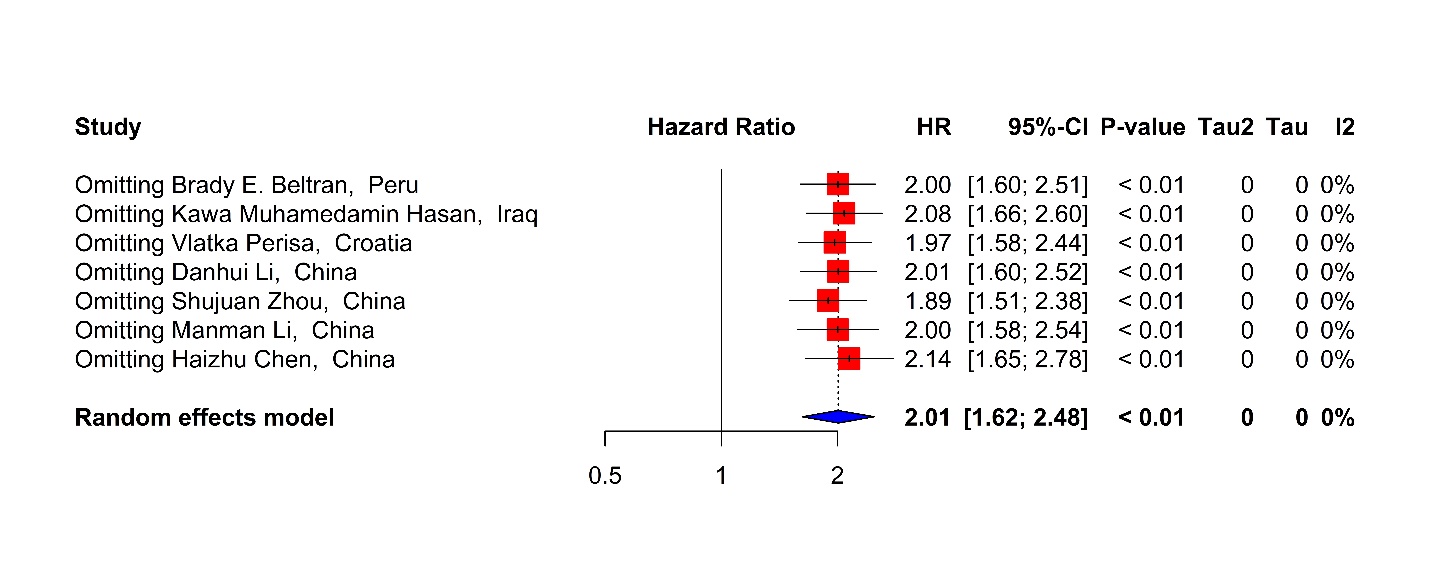


**Fig-1.** Sensitivity analysis in association RDW and OS


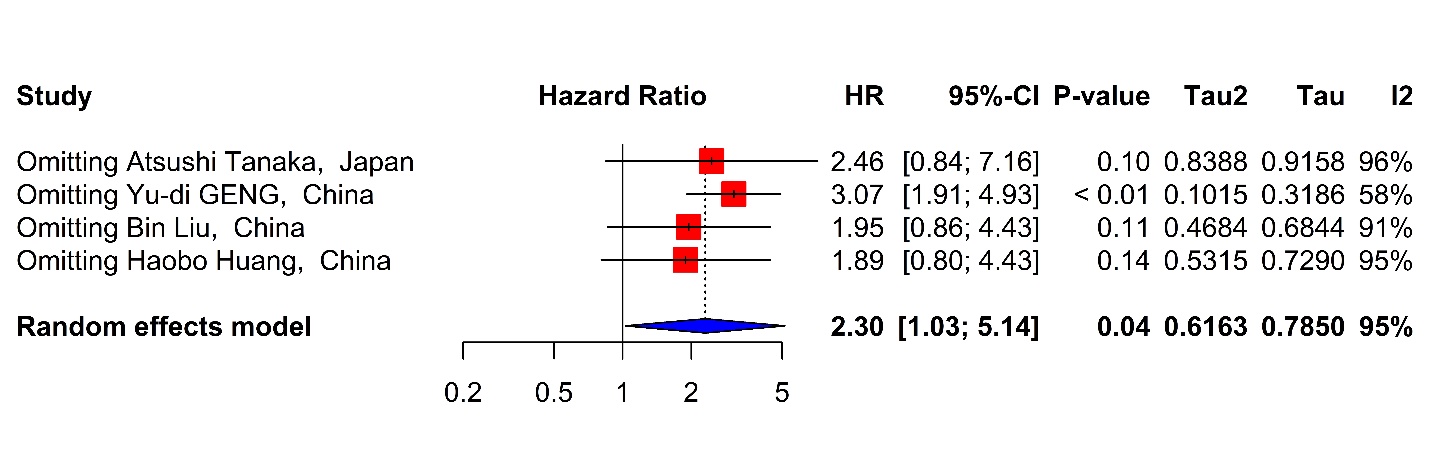


**Fig-2.** Sensitivity analysis in association of D-Dimer and OS


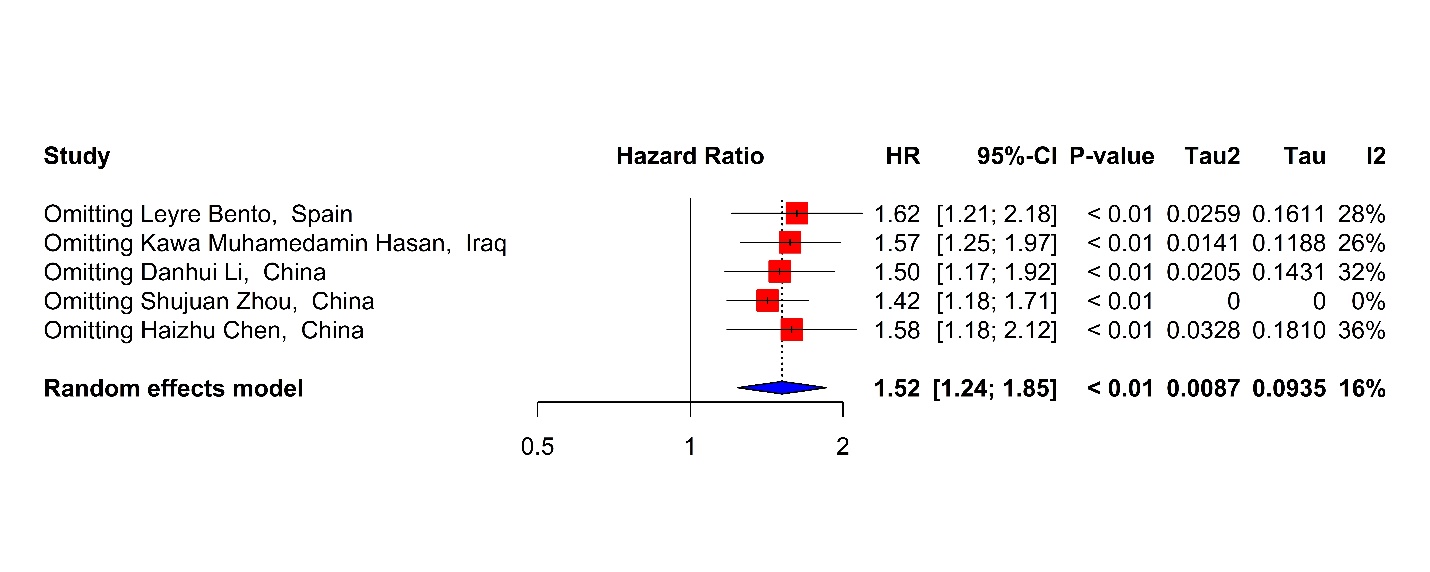


**Fig-3.** Sensitivity analysis in association of RDW and PFS
