# Supplementary material for: Prognostic value of red blood cell distribution width and D‐Dimer in diffuse large B‐cell lymphoma: Systematic review and meta‐analysis
Source: Cancer Rep (Hoboken). 2023 Nov 24;7(1):e1936. doi: 10.1002/cnr2.1936 (PMC10809198; doi:10.1002/cnr2.1936)
Supplement: Supplementary file 2 — Table 1 Quality assessments of studies include in meta‐analysis. [file CNR2-7-e1936-s001.docx]

**Table 1-** quality assessments of studies include in meta-analysis

| selection | | | | Comparability | outcome | | |  |  |
| --- | --- | --- | --- | --- | --- | --- | --- | --- | --- |
| Cohort studies | Representativeness of the exposed cohort | Selection of the non-exposed cohort | Ascertainment of exposure | Outcome was not present as baseline | *Control for most important factor and*  *Control for any additional factor* | Assessment of outcome | Adequate follow-up period for outcome | Adequacy of follow up of cohorts | score |
| Atsushi Tanaka (1) | a(1) | a (1) | a (1) | a(1) | a , b (2) | b(1) | a (1) | b (1) | 9 |
| Yu-di GENG (2) | a(1) | a (1) | a (1) | a(1) | a (1) | a (1) | b (0) | b (1) | 7 |
| Shaobo Duan (3) | a(1) | a (1) | a (1) | a(1) | a , b (2) | a (1) | a (1) | d (0) | 8 |
| Bin Liu (4) | a(1) | a(1) | a(1) | a (1) | a , b (2) | a (1) | a (1) | a (1) | 9 |
| Haobo Huang (5) | a(1) | a(1) | a(1) | a(1) | a , b (2) | a(1) | a (1) | a (1) | 9 |
| Brady E. Beltran (6) | a(1) | a(1) | a(1) | a(1) | a , b (2) | a(1) | a(1) | b(1) | 9 |
| Leyre Bento (7) | a(1) | a(1) | a(1) | a(1) | a , b (2) | b(1) | a (1) | a (1) | 9 |
| Kawa Muhamedamin Hasan (8) | a(1) | a(1) | a(1) | a(1) | a , b (2) | a(1) | a (1) | d (0) | 8 |
| Vlatka Periša (9) | a(1) | a(1) | a(1) | a(1) | a , b (2) | b(1) | a (1) | b (1) | 9 |
| Danhui Li (10) | b(1) | a(1) | a(1) | a(1) | a (1) | a(1) | a (1) | a (1) | 8 |
| Shujuan Zhou (11) | a(1) | a(1) | a(1) | a(1) | a , b (2) | a(1) | a (1) | b (1) | 9 |
| Manman Li (12) | a(1) | a(1) | a(1) | a(1) | a , b (2) | a(1) | a (1) | a (1) | 9 |
| Haizhu Chen (13) | b(1) | a(1) | a(1) | a(1) | b (1) | a(1) | a (1) | a (1) | 8 |

1. Tanaka A, Shimomura Y, Yabushita T, Ishikawa T. High Pretreatment Plasma D-Dimer Levels Are Associated with Poor Prognosis in Patients with Newly Diagnosed Diffuse Large B-Cell Lymphomas Treated with Immunochemotherapy. Blood. 2018;132:4219.

2. Geng Y-d, Chen Y-r, Jin J, Wang X-d, Zhang S, Li D-j. Prognostic value of D-dimer in patients with diffuse large B-cell lymphoma: a retrospective study. Current Medical Science. 2019;39:222-7.

3. Duan S, Zhang Y, Xu S, Jiang P, Qi Q. Contrast-Enhanced Ultrasound Parameters and D-Dimer: New Prognostic Parameters for Diffuse Large B-Cell Lymphoma. Cancer Management and Research. 2022:2535-44.

4. Liu B, Li B, Zhou P, Yue W, Wang T, Wang J, et al. Prognostic value of pretreatment plasma D-dimer levels in patients with diffuse large B cell lymphoma (DLBCL). Clinica Chimica Acta. 2018;482:191-8.

5. Huang H, Fan L, Fu D, Lin Q, Shen J. High pretreatment plasma D-dimer levels predict poor survival in patients with diffuse large B-cell lymphoma in the real world. Translational Cancer Research. 2021;10(4):1723.

6. Beltran BE, Paredes S, Castro D, Cotrina E, Sotomayor EM, Castillo JJ. High red cell distribution width is an adverse predictive and prognostic factor in patients with diffuse large B-Cell lymphoma treated with chemoimmunotherapy. Clinical Lymphoma Myeloma and Leukemia. 2019;19(9):e551-e7.

7. Bento L, Díaz‐López A, Barranco G, Martín‐Moreno AM, Baile M, Martín A, et al. New prognosis score including absolute lymphocyte/monocyte ratio, red blood cell distribution width and beta‐2 microglobulin in patients with diffuse large B‐cell lymphoma treated with R‐CHOP: Spanish Lymphoma Group Experience (GELTAMO). British journal of haematology. 2020;188(6):888-97.

8. Hasan KM, Elmeshhedany AY. Prognostic value of absolute lymphocyte/monocyte ratio, red cell distribution width and neutrophil/lymphocyte ratio in diffuse large B-cell lymphoma patients. Cellular and Molecular Biology. 2021;67(3):61-8.

9. Periša V, Zibar L, Sinčić-Petričević J, Knezović A, Periša I, Barbić J. Red blood cell distribution width as a simple negative prognostic factor in patients with diffuse large B-cell lymphoma: a retrospective study. Croatian medical journal. 2015;56(4):334-43.

10. Li D, Li S, Xia Z, Cao J, Zhang J, Chen B, et al. Prognostic significance of pretreatment red blood cell distribution width in primary diffuse large B-cell lymphoma of the central nervous system for 3P medical approaches in multiple cohorts. EPMA Journal. 2022;13(3):499-517.

11. Zhou S, Fang F, Chen H, Zhang W, Chen Y, Shi Y, et al. Prognostic significance of the red blood cell distribution width in diffuse large B-cell lymphoma patients. Oncotarget. 2017;8(25):40724.

12. Li M, Xia H, Zheng H, Li Y, Liu J, Hu L, et al. Red blood cell distribution width and platelet counts are independent prognostic factors and improve the predictive ability of IPI score in diffuse large B-cell lymphoma patients. BMC cancer. 2019;19:1-11.

13. Chen H, Zhong Q, Zhou Y, Qin Y, Yang J, Liu P, et al. Enhancement of the International prognostic index with β2-microglobulin, platelet count and red blood cell distribution width: a new prognostic model for diffuse large B-cell lymphoma in the rituximab era. BMC cancer. 2022;22(1):583.
